# Targeting the next generation of gamblers? Gambling sponsorship of esports teams

**DOI:** 10.1093/pubmed/fdac167

**Published:** 2023-03-31

**Authors:** Blair Biggar, David Zendle, Heather Wardle

**Affiliations:** 1 Department of Sociology, University of Glasgow, Glasgow G12 8QQ, Scotland; 2 Department of Computer Science, University of York, North Yorkshire YO10 5DD, UK; 3 Department of Sociology, University of Glasgow, Glasgow G12 8QQ, Scotland

**Keywords:** public health, young people

## Abstract

**Background:**

Esports fans are a target audience for gambling companies wanting to attract the next generation of bettors to their products. As with other sports, professional esports teams have commercial sponsorship arrangements. Our paper seeks to document the level of gambling sponsorship of the world’s top esports teams.

**Methods:**

A systematic review of the commercial arrangements between the top 20 esports clubs across three of the most followed esports titles was undertaken: Dota 2, League of Legends (LoL) and CS:GO. Data were scraped online relating to the top 20 teams competing at their respective major tournaments between October and November 2021.

**Results:**

Half of the esports teams in Dota 2 and CS:GO’s world championship events in 2021 were sponsored by gambling companies. Teams sponsored by gambling companies have a combined total of 25 868 912 followers across three major social media channels. No LoL teams were sponsored by gambling companies, yet sibling teams within the CS:GO and Dota 2 competitions were.

**Conclusion:**

The relationship between gambling companies’ sponsorship in esports should be considered in line with the calls for change in the relationship between football and gambling with gambling as a public health issue at its heart.

## Introduction

### Esports

Professional esports has grown in popularity and is now a multi-million-pound industry.^1^ Like other sports, there are different leagues for different formats of games, high-profile teams, sponsorship and advertising for both teams and competitions, merchandising and strong fandom and spectatorship. In 2021, it is estimated that the final of the Dota 2 world championship (The International10) had a peak audience of 2.7 million people,^2^ whereas the League of Legends (LoL) World Championship attracted around 4 million viewers.^3^ Prior work has suggested that esports fans tend to be a younger age demographic^4,5^ and have been described by gambling industry executives as the ‘wagerers of the future’.^6^

The increasing popularity of esports comes at a time when some traditional gambling companies are increasingly worried about the potential lack of interest in their products among Generation Z (broadly those born in the mid-to-late 1990s through to 2010).^6^ Esports fans offer a large target audience to focus marketing and offer products, both betting on esports and other forms of gambling.^6^ This relationship may already be being borne out in the academic literature: esports spectatorship appears to share a positive correlation with gambling involvement.^7,8^

Although increasing attention has been focused on the relationship between gambling companies and football,^9–11^ notable partnerships between gambling firms and esports have also been created. Much like in football, many professional esports teams have a roster (squad) of players who play in tournaments (for their game of expertise) of varying sizes across the world. Between competitions, teams train together. As esports teams have grown in profile, and prize pools for competitions have grown, so too have the teams of staff surrounding the players. The big teams now have coaches and managers for both gameplay and the business side of the team. They also have nutritionists and psychologists concerned with the health and success of the team. Some teams are known to host boot camps for young players to spot and nurture talent and potential future team players. Thus, esports teams are developing similar commercial models as teams in other sports such as football. This includes sponsorship and marketing partnerships. Teams wear strips and tracksuits for competitions and appear in branded advertising campaigns throughout the year. These campaigns are often based on the growth of the team’s brand but also the advertisement of their sponsors.

In the past few years, there has been growing attention to the rise in gambling sponsorship within football. Studies have found that this promotes, normalises and encourages gambling among football fans.^9^ In particular, the impact this has on young people should be noted. In the UK, 7% of children (aged 11–16) exposed to advertising and sponsorship noted that it prompted them to gamble when they were not otherwise going to do so.^12^ Among emerging adults and regular sports bettors, advertising and sponsorship prompted 87% of those experiencing problem gambling to gamble when they were not otherwise going to do so.^13^ In addition, lived experience groups in the UK and elsewhere have highlighted issues relating to sports sponsorship by gambling companies and sought to pressure a change in legislation (e.g.the Big Step campaign^14^). Key to the argumentation behind these campaigns is the normalisation and the promotion of gambling which for some is a health-harming activity.

Although these sponsorship arrangements between high-profile football clubs and gambling sponsors have attracted academic and policy attention, systematic focus on these arrangements in esports is nascent. Attention has been paid to the correlates of esports betting, and the characteristics of esports bettors, but to our knowledge, a systematic assessment of the extent to which gambling companies are involved in sponsoring and developing brand partnerships with esports clubs has not been undertaken.^15–17^ This paper fills this gap by presenting findings from a systematic review of the commercial arrangements between the top 20 esports clubs across three different leagues: Dota 2, LoL and CS: GO. Our findings document the level of gambling sponsorship of the world’s top esports team.

## Methods

To examine the commercial arrangements between betting companies and esports, we first selected three of the most popular esports title, based on viewership.^18–22^ We focused on Multiplayer Online Battle Arena games as they are played professionally by teams with rosters of players. As a result, they are more closely related to football teams that have squads of players wearing strips. For these reasons, we focused our analysis on Dota 2, CS: GO and LoL.

Across these three titles, different league structures exist, but all three have seasons that lead to an annual world championship (the season’s biggest tournament according to the prize pool and audience). The world championships are akin to football’s World Cup format, whereby teams look to qualify through different avenues according to rankings and eventually compete in a tournament over a few weeks. For Dota 2 and CS: GO, we chose the top 20 teams in each according to their ranking immediately before their respective world championship events. These rankings were set according to the teams’ performances in competitions in the lead-up to the world championships. The rankings were found on ESL Gaming’s website for Dota 2 and CS: GO alongside team information^1^. For LoL, we included the 22 teams that competed in the previous World Championships because up-to-date rankings were not available through the season.

Data were scraped from the internet between October 2021 and December 2021. During preparatory analysis of the data, we found that collecting data between October and December were optimal because the nature of sponsorship in the world of esports includes shorter-term deals, usually coinciding with the timing of the big tournaments. Some sponsorship deals may be advertised within the final weeks in the lead-up to the tournament and be for a period of a few months. Some others may be multi-year deals, usually with the biggest teams. Thus, our timing captured the status of commercial sponsorship deals at the time of the world championships (the Dota 2 International and the CS: GO Major Championships and LoL World championship or Worlds). The timing of our scraping was an attempt to capture the time in the year when teams were likely to have the most sponsors.

The process of scraping data of sponsorship was easier for some teams with a more established profile across social media and on their website. The main source of data for sponsorships and the most reliable was through teams’ official websites. These were cross-referenced with liquipedia.com, teams’ official social media accounts and relevant press releases that could be found through google. Using these tools, the following data were extracted: whether the team was sponsored by a gambling company, the name of the gambling company sponsoring, the start date of the deal and the team’s location. The location we include in Tables 1–4 is the place where the team is usually based. Although players (particularly during and after the pandemic) can be living in different locations globally from their teammates between competitions, the location is more about the base of the team as a corporate entity.

## Results

### Dota 2 analysis

The analysis of the Dota 2 teams shows that half of the top teams in the lead-up to TI in 2021 were sponsored by gambling companies which included shirt sponsorship. Thus, gambling companies’ branding could be viewed throughout the live streams of the tournament. Commonly, these sponsorship deals included the production of special branded content. Top teams, such as OG, can be seen across their online platforms in pictures and videos created for their sponsors. Gambling companies that sponsored teams included: Betway, Bitcasino.io, Parimatch, VBET, FUN88, Letou, Betway and Jing Ji Bao. Many of these brands offer both sports betting (including on esports) and online casino. Bitcasino is an online cryptocurrency casino, licensed in Curacao. It has been highlighted by Andrade, Sharman, Ziao and Newall (2022) that bitcasinos are riskier than other gambling operators such as Betway due to the nature of using cryptocurrency to gamble and the poor levels of consumer protection on crypto operators.^23^

Generally, teams were based in countries where gambling is legalised or is becoming so. PGS.LD was the exception. It is located in China, where commercial forms of gambling are not legal. PSG.LD is the esports company created by Paris Saint Germaine (PSG), a football giant owned by Qatar investment. PSG is sponsored by UNIBET, whereas its esports counterparts in Dota 2 are sponsored by Betway. In the case of their esports sponsorship, PSG.LGD are an example of a team with a major gambling sponsor despite the team’s location being a country where gambling is illegal.

It is also important to note the ‘other sponsors’ column. We see a significant number of endemic sponsors and large brands such as BMW and Nike. We also see other unhealthy commodities such as alcohol and energy drinks. In addition, there are several crypto and trading sponsors, such as crytpo.com and icmarkets. Despite not being gambling companies, these can represent gambling-like products.^20^ Like gambling, these products are often ‘gamified’ and come with a significant risk of harm due to the risk of users losing their investments.^20^ This is another area of overlap in this study with what has been observed in football.^24^

### CS:GO analysis

Like Dota 2, half (*n* = 10) of the top 20 CS:GO teams were sponsored by gambling companies with similar arrangements to those in Dota 2. Shirt sponsors were high, with branded online content across their social media channels. There is an overlap between the Dota 2 and CS:GO findings as some team brands compete in both leagues, and the sponsorship arrangement covers both leagues. Many of the gambling firms sponsoring CS:GO teams were similar to those sponsoring Dota 2 teams (Betway, Parimatch, Fun88 for example). Betway notably sponsored four different teams within the CS:GO league (Furia; BIG; G2; Ninjas in Pyjamas). Two of these deals were very recent, dating from 2021, showing the expansion of Betway in their sponsorship of esports. Although the teams Betway sponsored covered different jurisdictions, two were German-based teams.

### LoL analysis

LoL data suggested that there were not any LoL teams with clear sponsorship links to gambling companies. The explanation for this is clear. Unlike in Dota 2 and CS:GO, Riot Games, who developed LoL and have control over the World Championships and other big LoL tournaments, does not allow sponsorship by certain types of companies from appearing on players’ apparel. These include gambling, pornography and cryptocurrency exchanges.

## Discussion

### Main findings of this study

For Dota 2 and CS:GO in 2021, half (*n* = 10) of the top 20 teams also had gambling sponsorship. This shows the significant relationship between gambling companies and the world’s top esports teams in two of the biggest titles in esports: Dota 2 and CS:GO. Because of restrictions by LoL developers, Riot Games, the same pattern is not observed among LoL teams. However, restrictions within LoL did not mean that other rosters under the same banner could not be sponsored by these companies. For example, Betway is a sponsor of Unicorns of Love who appear here in our LoL table. However, this sponsorship appears only on the shirts of its CS:GO roster. This suggests that it is only the actions of the LoL developer that prohibits an even broader relationship between esports and gambling companies.

Interestingly, some companies sponsor several different teams within the same title—for example, Betway’s sponsorship of several CS:GO teams. Not all the teams sponsored were in geographically distinct markets (the equivalent of Betway sponsoring teams in the La Liga, The Premiership, etc.). Notably, Betway sponsored two separate German teams. More investigation is needed to explore these sponsorship motivations, but Betway’s sponsorship of two German teams may coincide with the widespread regulatory reform that is underway in Germany, permitting online gambling to be legalised for the first time.

It was also noticeable that some teams had gambling sponsors despite gambling not being legally available in the jurisdiction where the team is housed. This is similar to the sponsorship of British football teams by gambling operators who are not licensed in Great Britain. Here, the target audience is not the home audience, but rather the worldwide international audience and fanbase for these teams. It would seem similar processes are evident within esports.

### What is already known on this topic

In other sports, such as football, there is a considerable amount of research on the relationship between gambling companies and football. Research has highlighted that this both promotes and normalises gambling among football fans.^10^ Esports have an increasing revenue, viewership and status within mainstream entertainment.^6^ With an intense focus on betting companies’ sponsorship of football, and tightening of this in some jurisdictions, esports has been identified by the industry as an effective site to look for the gamblers of the future.^6^ Writing in 2021, Wardle described this process as a ‘quiet revolution’ within esports.^6^ This ‘quiet revolution’ has seen a growing number of gambling companies starting to target esports teams as they (and esports at large) have seen a growing audience and profile, alongside growing sponsorship, and prize pools. At the time of writing, Wardle reported that three of the top 10 esports teams, had gambling sponsors.^6^ This paper extends this analysis by systematically reviewing the sponsorship arrangements of the top teams competing in the world championships for three of the biggest esports titles Dota 2, LoL and CS: GO. The findings we have presented suggest that this quiet revolution has continued to grow.

The findings are important. Esports fans are thought to tend to be younger and disproportionately male. Indeed, a recent investigation of adolescent esports involvement found that approximately 20% of adolescents who had both gambled and played video games had also bet on esports.^16^ In Britain, a YouGov poll estimated that 70% of adult esports fans were aged 18–24.^25^ Playing games is less likely to predict whether individuals are active gamblers than if they watch esports.^7,8,26^ Rossi and Nairn (2021)^27^ argue that esports gambling content was especially appealing to those aged under 25 years, with 85% of followers of esports betting accounts on Twitter estimated to be under the age of 24.^28^ The same study of gambling advertising on Twitter found that 28% of engagement with esports gambling advertisements came from under 16-year olds.^29^ Esports are popular among younger adults and children, meaning greater levels of exposure to those who should be protected from advertising an age-gated product. This age group is most likely to experience gambling-related harms and thus increased exposure to gambling advertising is of concern. Preliminary studies on esports bettors suggest that they are mostly young men and from non-White ethnic backgrounds.^30,31^ These bettors are more likely to have problematic gambling behaviours and be heavy gamers.^30,31^ Thus, to target future gamblers through esports is also to target (particularly young) gamers who are already embedded within a whole ‘ecosystem … which may encourage or facilitate certain forms of gambling and gambling-like practices’.^6^

### What this study adds

To our knowledge, this is the first study to look systematically at the relationship between gambling sponsorship and esports teams. We show that gambling organisations are increasingly developing commercial sponsorship arrangements with esports teams and that these partnerships appear to have become increasingly popular over the last 2 to 3 years. Examining the commercial determinants of gambling harms requires focus on how the gambling industry promotes itself and its products. Much attention has been given to this with regards to football and gambling, but processes of equal magnitude are evident among esports teams. This is of particular concern, given the popularity of esports among children and the younger age demographic of its audience, and its impact should be monitored.

### Limitations of the study

Due to the lack of academic literature on esports, the paper was unable to draw heavily on trends in the existing academic literature on gambling and esports or esports sponsorship more broadly. As a result, this paper also represents a call for more attention to be given to (i) esports and (ii) its relationship with gambling companies more broadly. It would have been useful to have a record of the sponsorship to the same teams or of the competing teams at the same championships over the last few years as a means of comparison. However, these data were not available.

We believe that it will be important to track the progress of the relationship found here in further follow-up studies. Further research could also consider the wider area of esports by including/focusing on other popular esports with individual players such as Call of Duty or Fifa. Thus, there are multiple angles of this paper that should be explored further.

## Figures and Tables

**Table 1:** Summary table

*Title*	*Number of teams analysed*	*Number of teams with gambling sponsors*	*Total number of social media (Facebook, Twitter and Instagram) followers of teams with gambling sponsors. These aren’t mutually exclusive between titles as teams often have multiple competitive rosters*
Dota 2	20	10	13 254 731
CS: GO	20	10	12 614 181
LoL	22	0	N/A

**Table 2:** Top Dota 2 teams and sponsors

*Team*	*Location*	*Gambling Sponsor?*	*Date of gambling sponsorship*	*Other sponsors*
PSG.LGD	China	Betway	2018	Monster Energy; Doutu; HLA Jeans; Clear; Ipason; China; Citic Bank; Voltaren
T1	South Korea	No	N/A	Nike; BMW; Douyu; Red Bull; Samsung Odyssey; SKT 5GX; twitch; Hana Bank; Klevv; SteelSeries; Matrix Keyboards; Secretlab
Vici Gaming	China	No	N/A	Bixin; Voltaren; Douyu
Team Aster	China	No	N/A	Huya; FMWH; DXRacer; CHERRY
Evil Geniuses	USA (Seattle)	Bitcasino.io	2021	Wolverhampton Wanderers; Monster; LG Ultragear; Coinbase; Absolut; Bud Light; Elysian Brewing; Twitch; Secretlab; TUMI; PEAK6; POINT3
Team Spirit	Russia	Parimatch	2019 (renewed 2021)	Nike; Redbull; MyCSGO; HyperX
Invictus Gaming	China	Betway	2018	Huya TV; Chevrolet; Bixin; WYWK; Mirinda; Lilbetter; Secret Lab; Zeiss; Voltaren
Tundra Gaming	UK	No	N/A	Noble Chairs; Kappa; Tik Tok; PhoneCases3D; Adamas Esports
Virtus.Pro	Russia	Parimatch	2018 (extended in 2020 and 2021)	HyperX; Haval; Bybit; Kingston FURY
Alliance	Sweden	VBET	N/2021	Team Razer; Monster; Twitch; Newzoo; Socios.com; Bybit; Voodoo
Team Nigma	Europe/UAE	No	N/A	Twofour54 Abu Dhabi; Eithad Airways
Quincy Crew	USA	No	N/A	Since March 2020 they are independent of organisational sponsorship.
Elephant	China	No	N/A	No sponsorship
Royal Never Give Up	China	No	N/A	Kappa
TNC Predator	Philippines	No	N/A	Acer Predator; TheNet.com; DXRacer
Team Liquid	Netherlands/USA	No	N/A	Verizon; Alienware; Monster; SAP; Honda; IMC; Twitch; Jersey Mike’s Subs; Kingston; HyperX; HUYA; Secretlab
OG	Portugal	FUN88	2020	Red Bull; BMW; Steelseries; Socios.com; Secretlab; DMScript; ICMarkets
Fnatic	UK	Letou	2019 (renewed 2021)	BMW; crypto.com; JackLinks; Hisense; Letou.com; anda seat; Kaspersky; PC Specialist; ASOS; Monster
Beast Coast	USA/Peru	Betway	2020	HyperX; Aorus; Techstars; summaforte; Keeper; Trovo
Team Secret	Europe	Jingjibao (JJB.one)	2020	Corsair; Uniswap; Secretlab; TUMI; Predator; Maxis; Geforce Esports; Levante Brewing Company; DM Script; HUYA

**Table 3:** Top CSGO teams and sponsors

*Team*	*Location*	*Gambling Sponsor?*	*Date of gambling sponsorship*	*Other sponsors*
Natus Vincere	Ukraine	GG.Bet	2017–2020. Contract ended in 2020 and new deal struck in January 2021	Monster; Logitech; Raid: Shadow Legends; Socios.com; Puma; Tinkoff; Newzoo; Phillips; Esport.com; Huya.com; CS Money; Nvidia
Gambit Esports	Russia	Liga Stavok	June 2021 replaced VulkanBet	MTS; GGDrop; Ultragear; Abios; WASD.TV
Team Vitality	France	No	N/A	Orange; Adidas; Aldi; Corsair; Afflelou; Phillips; Quersus; Scuf Gaming
Ninjas in Pyjamas	Sweden	Betway	2017 and extended multiple times. Cancelled for Riot Games-related games in 2018	Team Razer; Tibber; Zilliqa
G2 Esports	Germany	Betway	2021 (2 year deal)	BMW; Logitech; Red bull; Bondly; Adidas; Agon by AOC; Philips; Pringles; Mastercard; Secretlab; AORUS; Domino’s Pizza Germany; Aimlab; Twitch; Paysafecard; Legion by Lenovo; Seasonic; Ralph Lauren
Heroic	Denmark	No	N/A	N/A
FaZe Clan	USA	No	N/A	G Fuel; McDonald’s; Scuf Gaming; Steel Series; HTC Gaming; wix.com; Manchester City; CTRL; Verizon
Astralis	Denmark	No	N/A	Bybiy; Logitech; Secret Lab; Omen; Audi; Power; Esportal; Cult; Lunar; Hummel; Capgemini; Bo & Olufsen; garmin; Amnesty International
OG	Portugal	FUN88	2020	Red bull; BMW; Steelseries; Socios.com; SecretLab; DMScript; ICMarkets
Team Liquid	Netherlands/USA	No	N/A	Verizon; Alienware; Monster; SAP; Honda; IMC; Twitch; Jersey Mike’s Subs; Kingston; HyperX; HUYA; Secretlab
Virtus.pro	Russia	Parimatch	2018 (extended in 2020 and 2021)	HyperX; Haval; Bybit
BIG (Berlin International Gaming)	Germany	Betway	2019 (2 year deal was extended in 2021)	HyperX; Haval; Bybit; Kingston FURY; omen; Skin Baron; Backford; Corsair; Coinbase; Die Bayerische; Volvic; Leet Desk; Wechsel Pilot
ENCE	Finland	No	N/A	Telia; Wolt; Red bull; Noble Chairs; Coinmotion; Logitech; LOIHDE Factor
FURIA Esports	Brazil/USA	Betway and Pokerstars	2021 – Betway; Pokerstars—2020	Red bull; Nike; Santander; AOC; HyperX; Twitch;
Team Spirit	Russia	Parimatch	2019 (renewed 2021)	Nike; Redbull; MyCSGO; HyperX
mousesports	Germany	No	N/A	Team Razer; Puma; Noble Chairs; Nitrado
Entropiq	Czech Republic	No	N/A	Puma; McDonald’s; your Gate; HAL3000; Ematiq; Fine Gusto
Complexity	USA	No	N/A	Twich; Miller Lite; Herman Miller; Nations; US Army; Dairy Max; Extra Life
Dignitas	USA	VIE	2019 (deal expanded in 2020)	SIG Susquehanna; Verizon; NYX Professional Makeu; QNTMPAY; Hyperx; Voodoo Range; US Air Force; Respawn; Zytara Labs
MAD Lions	Spain	No	N/A	SEAT Imagin; EPOS; Team Razer; GLS; Warner Music Spain; Kappa; Red Cross; AOC

**Table 4:** Top LoL teams and sponsors

*Team*	*Location*	*Gambling Sponsor?*	*Date of gambling sponsorship*	*Other sponsors*
DWG KIA	Korea	No	N/A	Kia Motors; Adidas; Logitech G; Hanatour; Zinus; Glocalize
Suning	China	No	N/A	OnePlus; Skyworth; Suning; KFC; Logitech G; HUYA
G2 Esports	Germany	No	N/A	BMW; Logitech; Red Bull; Adidas; Betway (but not for LoL); Agon by AOC; Philips; Pringles; Mastercard; Secretlab; AORUS; Domino’s Pizza Germany; Aimlab; Twitch; Paysafecard; Legion by Lenovo; Seasonic; Ralph Lauren; Bondly
FunPlus Phoenix	China	No	N/A	Scream; Bixin; L’Oreal Expert; BMW; OPPO; Herman Miller; Fish Cool
EDG	China	No	N/A	TCL; Bixin; Penguin Esports; Intel; Team Razer
RNG	China	No	N/A	Not found
LNG	China	No	N/A	Team Razer; LYNK&CO; Li-Ning
Gen.G	South Korea	No	N/A	LG; University of Kentucky; Eastern Michigan University; Mercedes-Benz; Monster; Puma; Douyu.com; Roccat; US Bank; Mcdonald’s; SIDIZ; Silicon Valley Bank
T1	South Korea	No	N/A	Nike; BMW; Douyul Red Bull; Red Ball; Samsung Odyssey; SKT 5GX; Twitch; Hana Bank; Klevv; SteelSeries; Matrix Keyboards; Secretlab
Hanwha Life	South Korea	No	N/A	Life Plus; Team Razer; douyu.com; Health Balance; KOLONFNC
Mad Lions	Spain	No	N/A	GLS; AOC; Warner Music Spain; Kappa; GOIKO; SEAT; Imagin; EPOS; Team Razer
Fnatic	UK	No	N/A	BMW; crypto.com; JackLinks; Hisense; Letou.com; anda seat; Kaspersky; PC Specialist; ASOS; Monster
Rogue	USA/Europe	No	N/A	Find your grind; Rekt Global; Autofull
100 Thieves	USA	No	N/A	Cash App; AT&T; JBL; Dollar Shave; Truly Hard Seltzer; Twisted Tea; Chipotle; Rockstar; Grub Hub; Omen; Rocket mortgage; StockX; Lexus
Team Liquid	Netherlands/USA	No	N/A	Verizon; Alienware; Monster; SAP; Honda; IMC; Twitch; Jersey Mike’s Subs; Kingston; HyperX; HUYA; Secretlab
Cloud9	USA	No	N/A	AT&T; BMW; EPOS; HyperX; Kaiser Permanente; Kingston; Microsoft; Puma; Red Bull; Secret Labl Twitch
PSG Talon	Hong Kong	No	N/A	PSG Esports; View Gear; Recaro; Laorus; Twitch
Beyond Gaming	Taiwan	No	N/A	Mega Bank; Notorious; Infinite Power; AMD; Sades; Team Razer
Unicorns of Love	Germany	No	N/A	Not found
Infinity Esports	Costa Rica	No	N/A	BMW; Office Depot; Logitech; Tik Tok; 1NF1TE; Cougarl The Word Company
Galatasaray Esports	Turkey	No	N/A	Nike; SIXT; NEF; NesINE.com; GETIR; Turkish Airlines; TUNC Holding; Burger King; Medical Park; ULKER; Hard Line; HDI; Deniz Bank; Aroma; socios.com; maximum; agapass.com; Passolig; Diversey; Damat Tween; Sportoto.gov.tr
RED canids	Brazil	No	N/A	Warrior; GigaPro Technology
DetonatioN	Japan	No	N/A	AU; CTC; ED ON; Sharp; Logicool; XTEN; Openrec.tv; Gtune; Wild Dish; Meiji; CX Split; NIDEK; Sharp; Elecom; GEForce; ZOWIE; WD_Black; DXRacer; Note; G Gaming; Fukuske; Newzoo
Peace	Australia	No	N/A	Varmilo; BEATHS; Republic of Gamers

## Data Availability

The data underlying this article will be shared on reasonable request to the corresponding author.

## Footnotes

1 See for details on how rankings were calculated https://pro.eslgaming.com/worldranking/dota2/about/
